# Rapid Identification of Methicillin-Resistant Staphylococcus aureus Using MALDI-TOF MS and Machine Learning from over 20,000 Clinical Isolates

**DOI:** 10.1128/spectrum.00483-22

**Published:** 2022-03-16

**Authors:** Jiaxin Yu, Ni Tien, Yu-Ching Liu, Der-Yang Cho, Jia-Wen Chen, Yin-Tai Tsai, Yu-Chen Huang, Huei-Jen Chao, Chao-Jung Chen

**Affiliations:** a AI Innovation Center, China Medical University Hospital, Taichung City, Taiwan; b Department of Laboratory Medicine, China Medical University Hospital, Taichung City, Taiwan; c Department of Medical Laboratory Science and Biotechnology, China Medical University, Taichung City, Taiwan; d Graduate Institute of Integrated Medicine, China Medical University, Taichung City, Taiwan; e Proteomics Core Laboratory, Department of Medical Research, China Medical University Hospital, Taichung City, Taiwan; f Department of Neurosurgery, China Medical University Hospital, Taichung City, Taiwan; g Laboratory Medicine, Feng Yuan Hospital, Ministry of Health and Welfare, Taichung City, Taiwan; h Department of Laboratory Medicine, Taipei Medical University-Shuang Ho Hospital, Ministry of Health and Welfare, New Taipei City, Taiwan; i Department of Laboratory Medicine, National Taiwan University Hospital Yunlin Branch, Yunlin County, Taiwan; j Department of Laboratory Medicine, Hualien Tzu Chi Hospital, Buddhist Tzu Chi Medical Foundation, Hualien County, Taiwan; University of Arizona/Banner Health

**Keywords:** methicillin-resistant *Staphylococcus aureus*, drug resistance, MALDI–TOF MS, LC–MS, machine learning

## Abstract

Rapidly identifying methicillin-resistant Staphylococcus aureus (MRSA) with high integration in the current workflow is critical in clinical practices. We proposed a matrix-assisted laser desorption/ionization-time-of-flight mass spectrometry (MALDI–TOF MS)-based machine learning model for rapid MRSA prediction. The model was evaluated on a prospective test and four external clinical sites. For the data set comprising 20,359 clinical isolates, the area under the receiver operating curve of the classification model was 0.78 to 0.88. These results were further interpreted using shapely additive explanations and presented using the pseudogel method. The important MRSA feature, *m/z* 6,590 to 6,599, was identified as a UPF0337 protein SACOL1680 with a lower binding affinity or no docking results compared with UPF0337 protein SA1452, which is mainly detected in methicillin-susceptible S. aureus. Our MALDI–TOF MS-based machine learning model for rapid MRSA identification can be easily integrated into the current clinical workflows and can further support physicians in prescribing proper antibiotic treatments.

**IMPORTANCE** Over 20,000 clinical MSSA and MRSA isolates were collected to build a machine learning (ML) model to identify MSSA/MRSA and their markers. This model was tested across four external clinical sites to ensure the model’s usability. We report the first discovery and validation of MRSA markers on the largest scale of clinical MSSA and MRSA isolates collected to date, covering five different clinical sites. Our developed approach for the rapid identification of MSSA and MRSA can be highly integrated into the current workflows.

## INTRODUCTION

Methicillin-resistant Staphylococcus aureus (MRSA) has become a major health care concern worldwide because it resists most antibiotic treatments for ordinary staph infections. Rapid identification of MRSA can enable proper antimicrobial therapy and infection-control intervention by clinicians. Conventional methods usually require 12 h to 24 h of sample culture and an additional 24 h to 48 h for identifying the bacterial species and conducting antibiotic susceptibility testing (AST). Phenotypes are typically identified by disk diffusion, Epsilometer testing (E test) gradient disk diffusion, broth macro, and microdilution in the clinical microbiology laboratory (1). However, the aforementioned methods are laborious and their results can be affected by physiochemical factors such as nutrient media, pH, temperature, and solubility. Therefore, a microbial culture report requires 2 to 3 days to complete. In contrast, genotypic AST methods (e.g., polymerase chain reactions [PCRs], DNA microarrays, and DNA chips) eliminate the long incubation time and reduce the contamination risk. Although the mecA PCR method can rapidly identify MRSA, PCR tests are costly and their quality depends on the skill of the personnel. Furthermore, some coagulase-negative staphylococci and methicillin-sensitive S. aureus isolates, which contain the mecA gene, can be mistakenly identified as MRSA (2). Automated biochemical AST, such as bioMérieux ViTEK2 and BD Phoenix, can reduce hands-on time and turnaround time, and provide higher reproducibility. However, the automated biochemical AST has high cost and could not be affordable in small clinics.

The matrix-assisted laser desorption/ionization-time-of-flight (MALDI–TOF) technique has produced peptide/protein mass spectrometry (MS) fingerprints for searching mass-spectra libraries of bacterial species (3). Combined with commercial protein profiling databases (Biotyper MS or Vitek MS), MALDI–TOF is used for routine bacterial species identification in many hospitals. MALDI–TOF is recognized as a rapid, precise, low-cost, and less laborious method for bacterial species identification than conventional biochemical tests (4).

MALDI-TOF have been applied to detect phenol-soluble modulin (PSM) in MRSA (5), KPC-producing Klebsiella pneumoniae (6), cfiA positive/negative in Bacteroides fragilis (7), and to differentiate between Mycobacterium chimaera and Mycobacterium intracellulare (8). However, MALDI–TOF combined with the current database (Biotyper MS or Vitek MS) cannot identify bacteria that are resistant to multiple antibiotics. Therefore, MRSA must be identified by the above-mentioned AST methods (requiring approximately 18 h to 48 h).

To rapidly distinguish MRSA from methicillin-susceptible S. aureus (MSSA) without using AST methods, MALDI–TOF has recently been combined with machine learning (ML) methods for antibiotic-resistance prediction. Tang et al. discovered nine characteristic peaks of MRSA among 214 S. aureus isolates (9). Liu et al. discovered 38 characteristics peaks for a classification model from 452 clinical S. aureus isolates (10). Wang et al. processed 4,858 mass spectra using various ML methods and identified 200 peaks as marker attributes for a predictive model. Although they reported an area under the receiver operating curve (AUC) of 0.845, the generalization capability of their ML model may be limited since the model was tested within a same hospital system (11). Furthermore, because of bacterial diversity (12), it is essential to increase the source variability of clinical data to ensure the ML model robustness.

To this end, the present study proposes a robust model for rapid discrimination between MSSA and MRSA, for which isolates were collected from five clinical sites with retrospective and prospective tests to ensure ML model generalization. Furthermore, the model interpretability was enhanced using SHapely Additive exPlanations (SHAP) values (13). The most important feature marker was identified, and its possible role in methicillin resistance was investigated using molecular docking simulations.

## RESULTS

### Model performance of MRSA and MSSA.

**FIG 1:**
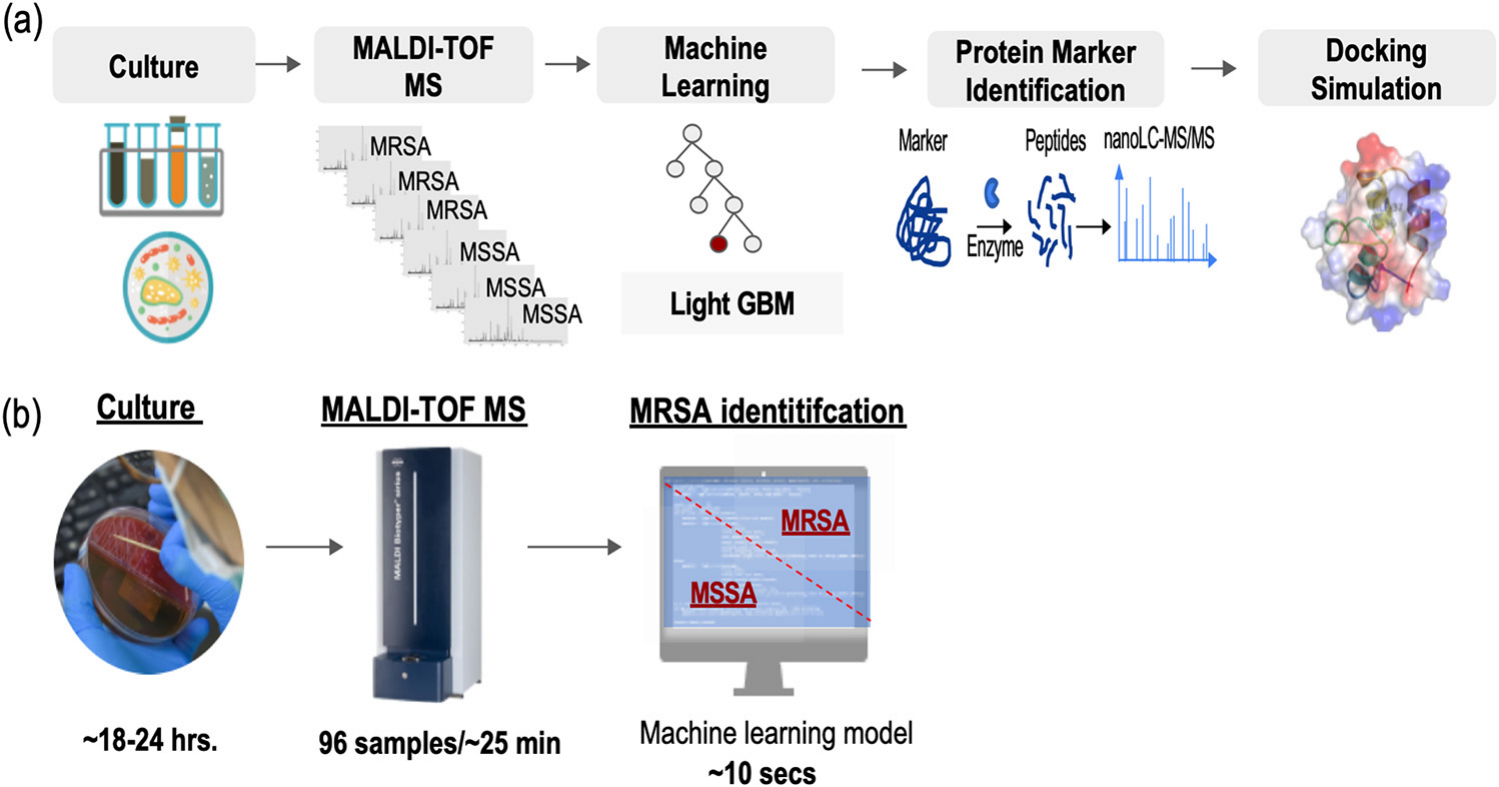
(a) Analytical flowchart of this study. The developing steps including clinical isolate culture, MALDI-TOF analysis, machine learning (ML) modeling and validation, protein marker identification, and docking simulation. (b) The time spent for sample culture, MALDI-TOF analysis, and MRSA determination by a ML model.

In this study, we used a large MALDI-TOF data set to develop a reliable ML model for rapid MRSA identification. For routine clinical practice, after MALDI-TOF analysis of cultured clinical strains, the MALDI-TOF spectra can be rapidly identified (within ∼10 s) as MRSA or MSSA using our ML model (Fig. 1b) instead of using conventional AST methods that spend ∼18 h to 48 h.

The MALDI–TOF 5802 spectra of the S. aureus isolates obtained from China Medical University Hospital (CMUH) were used for model training and internal validation. The MRSA and MSSA species were discriminated using LightGBM. Due to the bacterial diversity, we collected real-world clinical data from four external hospitals, representatives of North Taiwan (SSH), Middle Taiwan (FYH), South Taiwan (NTUH-YL), and East Taiwan (HTCH).

The model performances and data sizes at each clinical site are listed in Table 1. In a prospective study of approximately 2,500 isolates from January 2020 to June 2021, the AUC is 0.91. In another test of more than 12,000 isolates from external clinical sites from 2018 to 2020, the AUC ranged from 0.78 to 0.88. Our ML model shows high performance in the validation of four external clinical sites, indicating the model’s high precision and robustness.

**TABLE 1:** Trained model performances on CMUH MRSA samples from 2018 to 2019, CMUH samples from 2020 to 2021, and samples from four external clinical data sets

Site	Data segment	Sensitivity	Specificity	AUC	MRSA	MSSA	Total
CMUH	2018–2019 training	0.82	0.85	0.91	2,998	2,804	8,258
	2020–2021 prospective test	0.83	0.85	0.91	1,311	1,145	
FYH		0.83	0.65	0.78	3,200	2,206	5,406
NTUH-YL		0.72	0.88	0.88	1,116	1,012	2,128
SHH		0.74	0.80	0.85	1,876	1,292	3,168
HTCH		0.83	0.73	0.86	802	597	1,399

### Significant peaks of MRSA and MSSA.

**FIG 2:**
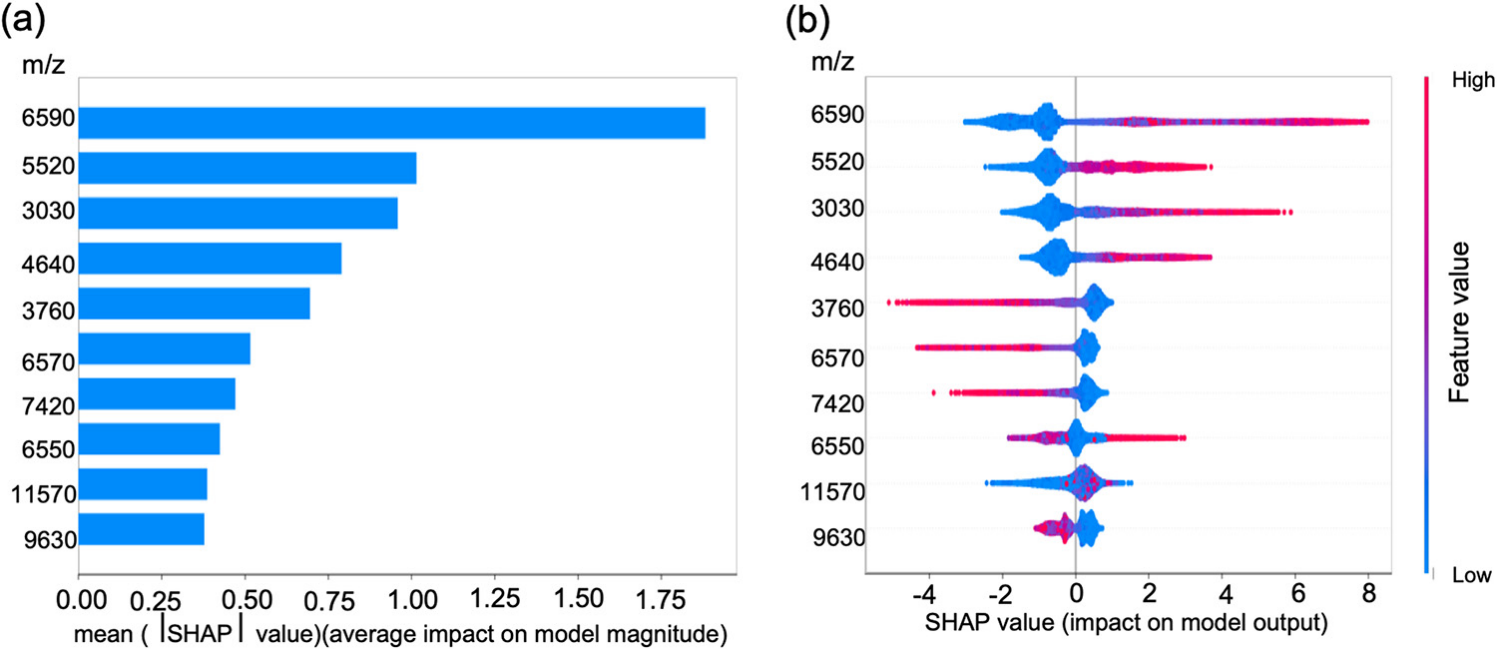
SHAP summary of the top 10 molecular features: (a) the highest-ranking feature (*m/z* 6,590 to 6,599) contributes more to the model prediction than the lower ones and has the highest predictive power; (b) positive and negative impacts of the top 10 molecular features in predicting MRSA.

To resolve the lower interpretability issue and discover the markers between MRSA and MSSA, the high-ranking molecular features in our training data set were identified using the SHAP method. SHAP is developed based on a game-theoretic approach to explain ML models by ranking the importance of molecular features in outcome prediction. The SHAP summary graph displays the 10 most important molecular features along the *y* axis (Fig. 2) and their impact along the *x* axis, where a more positive (negative) SHAP value indicates a higher impact of the molecular feature in classifying MRSA (MSSA). The top variables make higher contributions to the model prediction (i.e., have greater predictive power) than the bottom ones. Some of these top molecular features, such as *m/z* 6,590 to 6,599; *m/z* 5,520 to 5,529; *m/z* 3,030 to 3,039; *m/z* 3,760 to 3,769; and *m/z* 6,550 to 6,559 were observed as potential markers in previous studies, and were related to different clonal lineages (11, 14–17).

**FIG 3:**
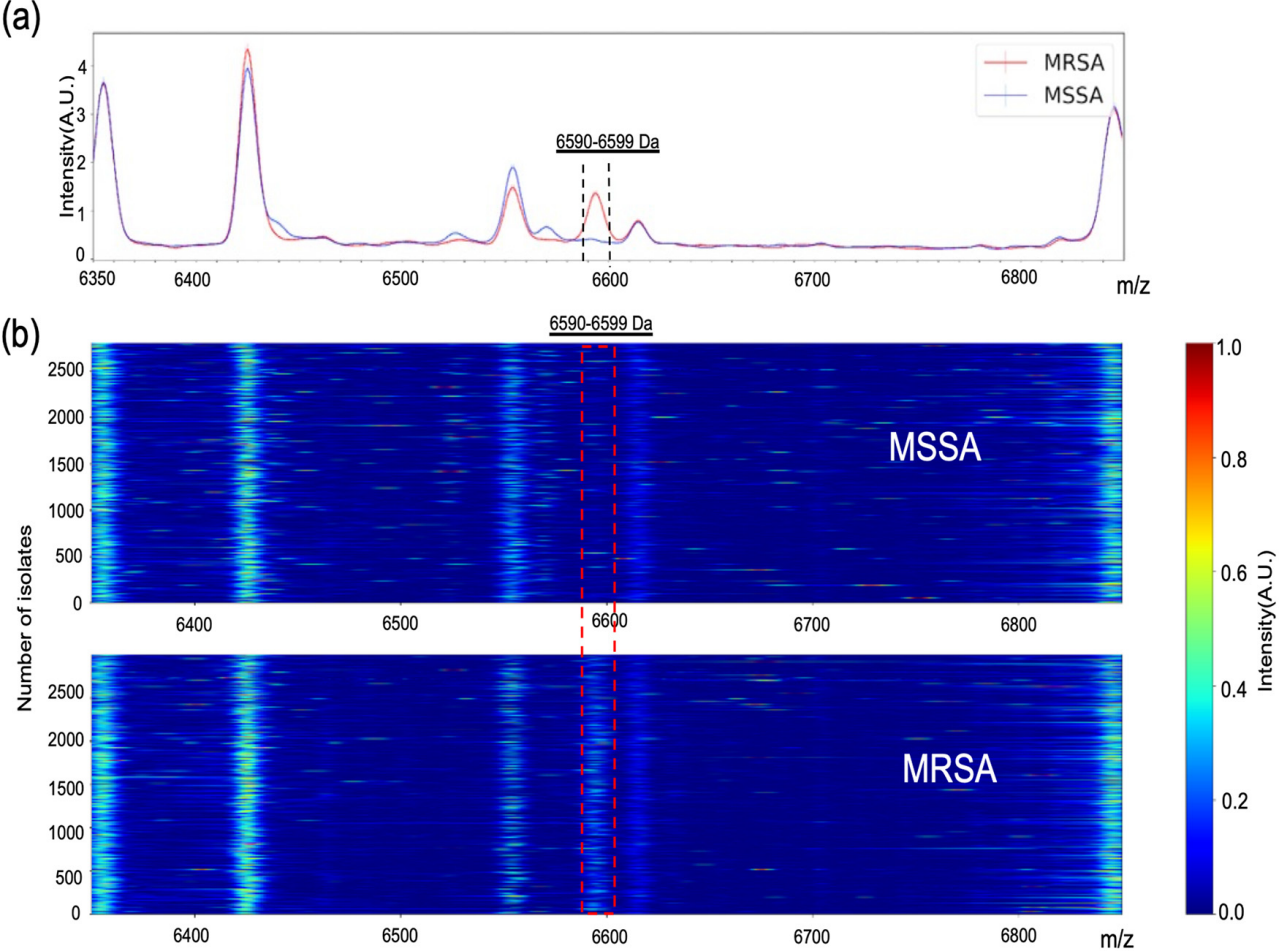
Two representations of the most important feature (6,590 to 6,599 Da) in the CMUH data set. (a) The average signals of all MALDI–TOF spectra measured from MSSA (plotted as blue lines.) and MRSA (plotted as red lines), respectively, was plotted using Python. (b) pseudogels of all individual samples; *y* axis represents the number of isolates, and *x* axis represents *m/z*. The MALDI-TOF pseudo-gel was plotted using Python.

**FIG 4:**
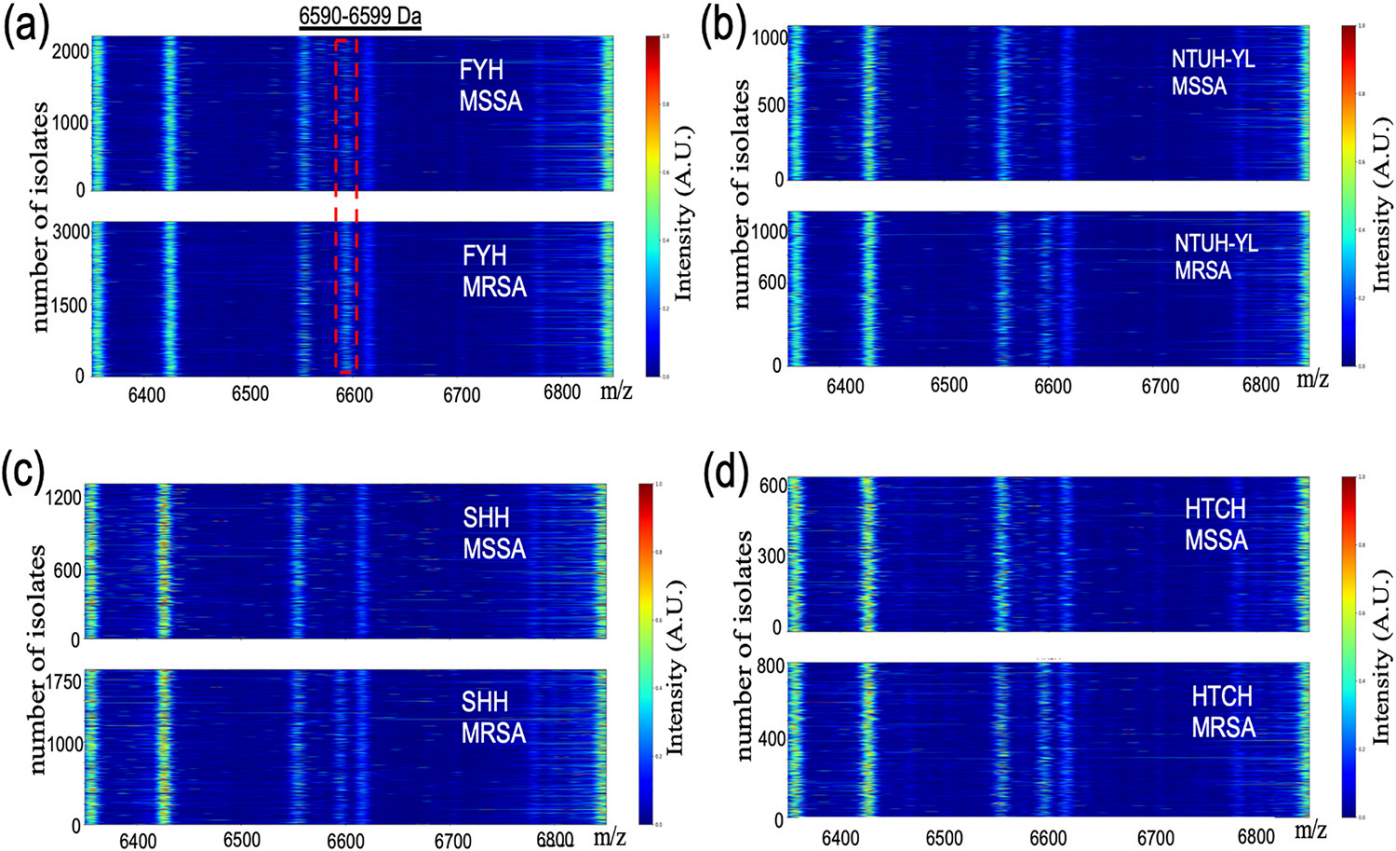
Observations of the molecular feature at 6,590 to 6,599 Da in the four external clinical data sets: (a) FYH, (b) NTUH-YL, (c) SHH, and (d) HTCH; *y* axis represents the number of isolates, and *x* axis represents *m/z*. We provide the first identification and visualization of a drug-resistant biomarker in a large clinical data set including four external clinical sites in retrospective and prospective tests.

The top-ranking molecular feature was the peak of *m/z* 6,590 to 6,599, indicating that this feature is the most important predictive feature in our data set. To better understand this feature, the differences between the MRSA and MSSA samples were revealed using our developed pseudogel visualization method (Fig. 3). The molecular feature of 6,590 to 6,599 Da appeared mainly in the MRSA diagram, affirming that this feature was the major discriminator between MRSA and MSSA in our model. Moreover, the molecular feature of 6590 to 6599 Da was constantly observed across the four external clinical data sets (Fig. 4), and a consistency pattern also been observed annually across 2018 to 2021 in CMUH data set (Fig. S1).

### Identification of the top-ranking protein marker of MRSA.

**FIG 5:**
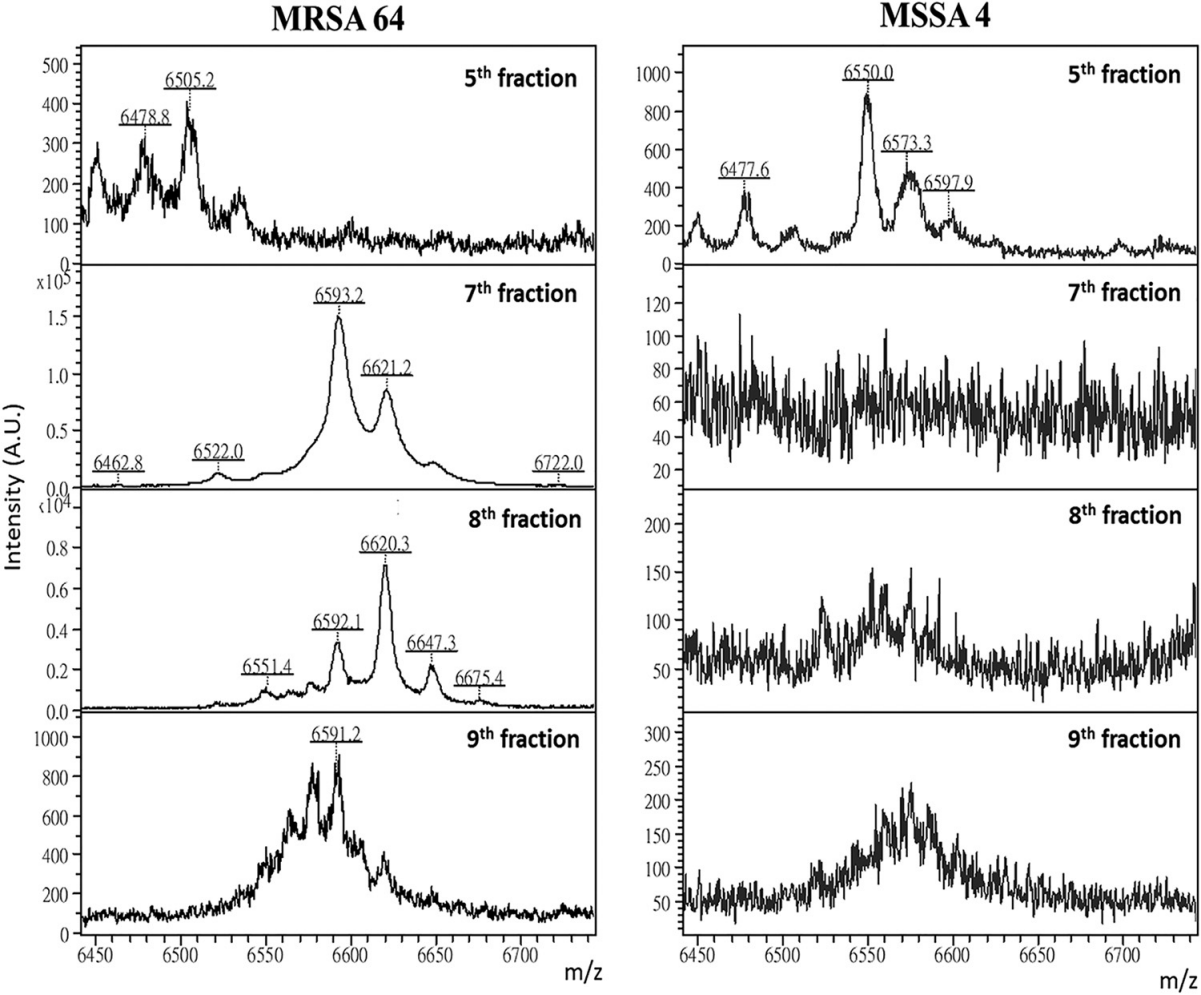
Mass spectra of MRSA64 and MSSA4 samples after fractionation on a C4 column. Shown are the mass spectra of the 5th, 7th, 8th, and 9th fractions. The *x* axis denotes the *m/z* values of the spectral signals (6,400 to 6,800).

To identify the protein sequence of *m/z* 6,590 to 6,599, the extracted protein solution from MRSA and MSSA strains was fractionated on a C4 column. Each fraction was collected and analyzed by MALDI–TOF MS to confirm the presence of the protein peak of *m/z* 6,590 to 6,599. The fractionation with protein peak markers was digested followed by nanoLC–MS/MS analysis and a protein database search. Fig. 5 shows the MALDI–TOF spectra of the collected fractionations from the representative MRSA and MSSA isolates. Comparing these mass spectra, we identified a significant peak signal of *m/z* 6,550.0 in the fifth fraction of the MSSA isolate, which was absent in the MRSA isolate. On the contrary, a peak signal of *m/z* 6,593.2 was detected in the seventh fraction of the MRSA isolate, but was absent in the MSSA isolate (Fig. 5). To have a more comprehensive sequence coverage, two enzymes of trypsin (cleave site: K and R) and Glu-C (cleave site: E) were separately used for target protein digestion. The seventh fraction separately collected from the MSSA and the MRSA isolate was digested and subjected to nanoLC-MS/MS analysis and mascot search. The peak of *m/z* 6,593.2 was identified as UPF0337 protein SACOL1680 (UniProt accession no. Q5HFD7) or UPF0337 protein SAUSA300_1582 (UniProt accession no. Q2FGA1) (Fig. S2, Table S2), because this protein was only identified in the seventh fraction from the MRSA isolate but not from the MSSA isolate. The fifth fraction separately collected from the MSSA and the MRSA isolate was also digested and subjected to nanoLC-MS/MS analysis and mascot search. The peak of *m/z* 6,550.0 was identified as UPF0337 protein SA1452 (UniProt accession no. Q7A593), because this protein was only identified in fifth fraction from the MSSA isolate but not from the MRSA isolate.

### Molecular docking simulation.

In the SHAP and pseudogel visualization methods, the important molecular feature at *m/z* 6,590 to 6,599 was identified as a digital biomarker that discriminated MRSA from MSSA. The protein-sequence results of nanoLC–MS/MS experiments showed that the UPF0337 protein SACOL1680 (*m/z* 6,590 to 6,599) of MRSA is a mutated form of the UPF0337 protein SA1452 (*m/z* 6,550 to 6,559) of MSSA. UPF0337 protein SA1452 and UPF0337 protein SACOL1680 belong to the CsbD family of proteins, considered as general stress-response proteins in bacteria (18).

However, there is no direct link between CsbD family protein and methicillin resistance. To pinpoint the possible underlying biological mechanism of drug resistance and CsbD family proteins, we performed protein–ligand docking simulations on methicillin between UPF0337 protein SA1452 (^36^AT^37^, *m/z* 6,550) of MSSA and UPF0337 protein SACOL1680 (^36^VI^37^, *m/z* 6,593) of MRSA.

**FIG 6:**
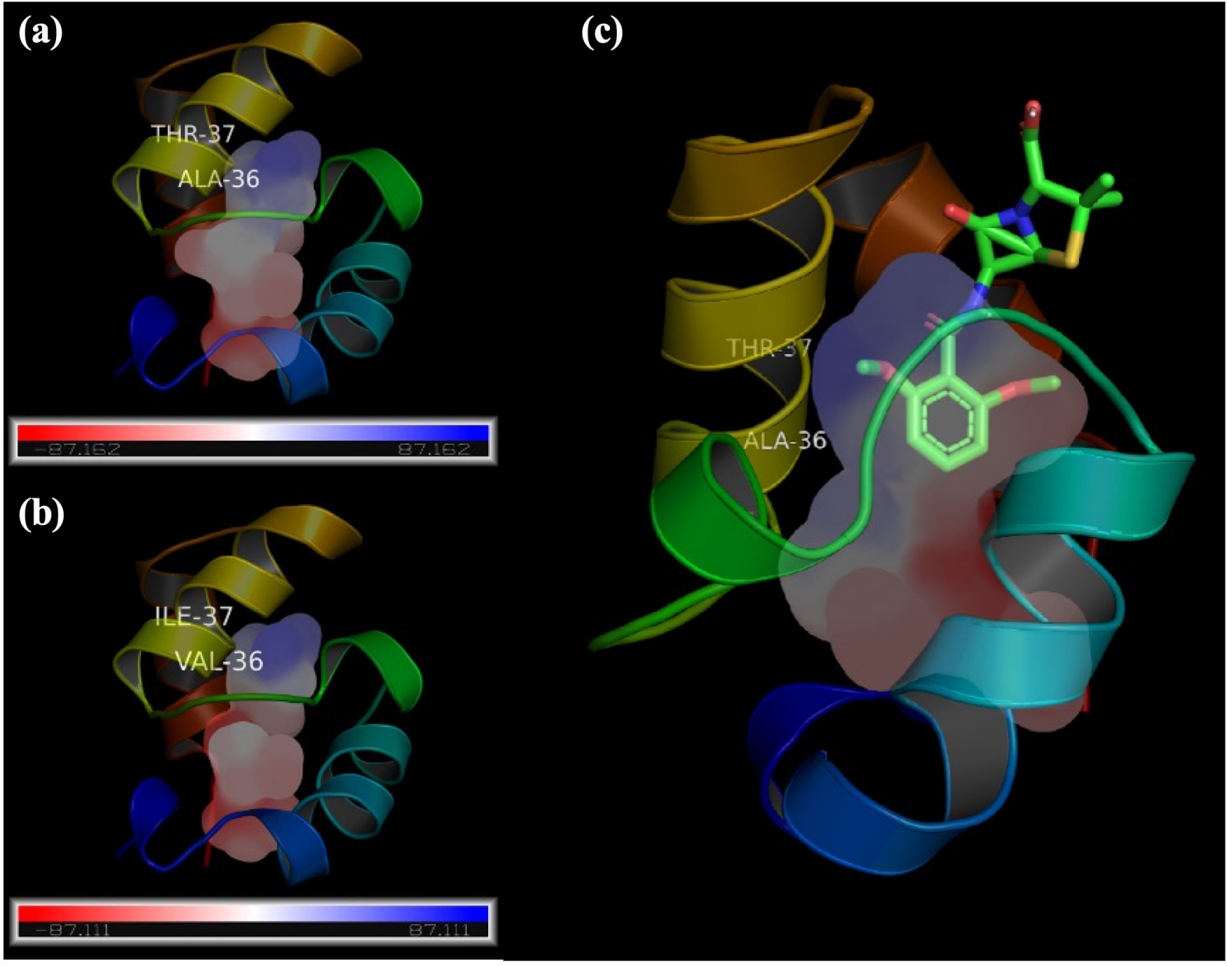
Protein–ligand docking simulation shows cavity difference between (a) ^36^AT^37^ (MSSA) and (b) ^36^VI^37^ (MRSA). When the binding area was limited within cavities, (c) the simulation result shows that methicillin can bind with ^36^AT^37^(MSSA) but not with ^36^VI^37^ (MRSA).

Phyre2 was adapted for protein-structure homology modeling (19). After generating the protein structures with the two different amino acids ^36^AT^37^ and ^36^VI^37^, protein–ligand docking simulations were performed in PyRX version 0.8 with PyMol for visualization. As shown in Fig. 6, after the two-amino-acid mutation, the ^36^VI^37^ cavity was smaller than the ^36^AT^37^ cavity. In the docking simulation, the best binding affinities of ^36^AT^37^-methicillin (MSSA) and ^36^VI^37^-methicilin (MRSA) were −5.4 and −4.9 kcal/mol, respectively. However, if the binding area was limited within the cavity, the binding affinity of ^36^AT^37^-methicillin (MSSA) was −5.3 kcal/mol and no docking result was observed for ^36^VI^37^-methicillin (MRSA). The above result may indicate that methicillin does not bind with UPF0337 protein SACOL1680 (^36^VI^37^, *m/z* 6,593), inducing a possible MRSA drug resistance.

## DISCUSSION

The machine learning approach is superior at finding mapping relationship, and maps peak features in a large data set of MALDI-TOF spectra with their corresponding antibiotics resistance in this study. To retain the completed peak information for machine learning training, we developed MALDI-TOF MS data process pipelines to extract completed peak information from the original raw data but not from the converted/exported peak file, which usually produces oversimplified and low-reproducible peak information. Furthermore, because different clinical sites come with different clinical data format of identification and antimicrobial susceptibility testing results in this study, we also build tools and pipelines for the automation of data processing, including regular expressions and text mining, to pair the clinical information of the I.D. numbers/AST results and their corresponding MALDI-TOF MS data. The data preparation and data cleaning ensured the MS data quality across five different clinical sites for further model training.

The performance of our ML model was somewhat less in NTUH-YL, SHH, and HTCH compared with CMUH. However, FYH has a significantly lower AUC value compared with four other clinical sites. The lower AUC in FYH is due to a lower specificity of 0.65, which means some strains were predicted as MRSA in our trained ML predicts but they were identified as MSSA in FYH AST reports. FYH used Vitek 2 instead of Phoenix for AST; however, Gargis et al. have reported that MRSA could be miscategorized into MSSA in Vitek 2 system (20), which could result in the lower specificity in our ML model when predicting MRSA using FYH data set.

Although the top-ranking protein marker of *m/z* 6,590 to 6,599 has been observed in other studies (7, 13), its protein sequence has not yet been identified with a reliable bottom-up proteomics method. The average neutral mass of UPF0337 protein SACOL1680 is *m/z* 6,722.51, but after degrading the first amino acid methionine (Met) (a loss of 131 Da), the protein average neutral mass is *m/z* 6,591.31, equaling the observed mass of 6,593.2 ± 3 Da in the MRSA isolate (Fig. 5). The peak signal in the fifth fraction of the MSSA isolate (*m/z* 6,550.0) was identified as UPF0337 protein SA1452 (UniProt accession no. Q7A593). The average neutral mass of UPF0337 protein SA1452 is *m/z* 6,682.41, but after degrading the first amino acid methionine (Met) (a loss of 131 Da), the protein average neutral mass is *m/z* 6,551.41, equaling the observed mass of 6,550.0 ± 3 Da in the MSSA isolate (Fig. 5). Comparing the protein sequences of the two similar UPF0337 proteins in MSSA and MRSA, we found that two amino acids in the MSSA isolates, ^36^AT^37^ of UPF0337 protein SA1452, were replaced with ^36^VI^37^ of UPF0337 protein SACOL1680 in the MRSA isolates. The protein mutation may induce a possible drug resistance because molecular docking simulation showed that methicillin can bind with UPF0337 protein SA1452 (^36^AT^37^, *m/z* 6,550) of MSSA but cannot bind with UPF0337 protein SACOL1680 (^36^VI^37^, *m/z* 6,593) of MRSA.

In conclusion, we collected over 20,000 clinical MSSA and MRSA isolates to build a ML model to identify MSSA/MRSA and discovered reliable markers with a possible drug-resistant property. We built data pipelines to collect completed peak information for machine learning, and data visualization tools were also built for better model interpretability. The model was retrospectively and prospectively tested across four external clinical sites to ensure the model’s usability. To the best of our knowledge, we report the first discovery and validation of MRSA markers on the largest scale of clinical MSSA and MRSA isolates collected to date, covering five different clinical sites. Our developed MALDI–TOF MS-based ML model for the rapid identification of MSSA and MRSA can be highly integrated into the current workflows of clinical services and can save valuable time for physicians seeking proper antibiotic treatment; however, this rapid screening assay cannot replace more sensitive and specific methods of MRSA detection.

## MATERIALS AND METHODS

The analytical flowchart is depicted in Fig. 1a. MSSA and MRSA isolates from clinical samples were cultured and analyzed with MALDI-TOF. The large size of MALDI-TOF data set were trained by ML and the model was validated across four clinical sites to ensure the robustness. The most important protein marker indicated in the ML model was identified with nanoLC-MS-MS. The possible role of the identified protein marker in methicillin resistance was investigated using molecular docking simulations. The detailed process of the above-mentioned steps is described as follows.

### Bacterial collection and data source.

Clinical samples were collected from blood, cerebrospinal fluid, ascites, pleural fluid, body fluid, sputum, tissue, bile, stool, urine, and other clinical specimens. The study included 8,258 cases (4,309 MRSA cases and 3,949 MSSA cases) at the China Medical University Hospital (CMUH) clinical microbiology laboratory from January 2018 to June 2021. The mass spectral data for the predictive model development were obtained retrospectively (IRB number: CMUH109-REC3-098). An additional 12,101 mass spectra collected during the 2018–2020 period was provided by other hospitals: Feng Yuan Hospital (FYH, IRB number: 110004), National Taiwan University Hospital Yunlin Branch (NTUH-YL, IRB number: 202101073W), Shuang Ho Hospital (SHH, IRB number: N202103122), and Hualien Tzu Chi Hospital (HTCH, IRB number: 110-053-B). These data were reserved for external validation of the predictive model’s performance. All MSSA and MRSA strains were confirmed by a Phoenix AST system (CMUH, NTUH-YL, SHH, HTCH.) and a VITEK2 AST system (FYH).

### Identification of S. aureus by MALDI–TOF.

A single colony of S. aureus on tryptone soya broth (TSB) agar plates was picked and smeared onto a spot on a MALDI target plate. The sample spot was overlaid with 1 μL of 70% formic acid (FA) and dried at room temperature. Another 1 μL of α-cyano-4-hydroxycinnamic acid (CHCA) matrix solution (10 mg/mL, 50% I/2.5% trifluoroacetic acid [TFA]) was added to the sample spot. Once the sample spot had dried, the MALDI plate was inserted in a MALDI–TOF mass spectrometer (Microflex LT, Bruker Daltonik, Germany) with FlexControl software (version 3.4) and MALDI Biotyper (MBT) Compass version 4.1 for analysis. Spectra were collected at *m/z* 2,000 to 20,000. The identification results were produced in MALDI Biotyper Realtime Classification. The sample preparation protocol, MALDI–TOF MS instrument and instrumental control software were all the same to generate the spectra in the five hospitals.

### MS data processing for ML.

The pipelines were built using Python version 3.7 for the data processing, including MALDI-TOF MS raw data extraction, signal processing, model training and data visualization. As the original sampling frequencies differed in some cases, the resolutions of all cases were resampled to a fixed sampling rate (1 Da in this study). TopHat, a square root transformation and Savitzky–Golay-filter were used for baseline correction, normalization and smoothing, respectively. After baseline correction and intensity normalization, the data in the temporal domain were converted to *m/z* ratios and the mass spectra within the range of 2,000 to 20,000 Da were extracted, and the following min-max scaling equation was performed to ensure signal intensity is in the range of 0 and 1.

$$x\text{′}=\frac{x-x_{\min}}{x_{\max}-x_{\min}}$$

To reduce peak shifting, the extracted spectra were grouped into bins. Peaks located within the same bin were considered as the same attribute. The peak intensities within each bin were averaged to obtain the mean intensity in that bin. After preprocessing all MALDI–TOF data of the S. aureus cases as described above, the labels (MRSA or MSSA) and the preprocessed MALDI–TOF data were fed into multiple ML algorithms for training and validation.

Furthermore, we fed five different bin sizes (1–Da, 5–Da, 10–Da, 15–Da, and 20–Da) into 10 ML algorithms (i.e., light gradient boosting machine [LightGBM], gradient boosting, logistic regression, extreme gradient boosting, extra trees, random forest, linear SVM, decision tree, K neighbors and naive Bayes) with 5-fold cross-validation, and the combination of bin size and ML algorithms was presented in Table S1. The best combination was selected according to the following F_1_ score equation:

F1= 2 True positive2 True positive + False positive + False negative

In the study, we applied 10–Da bin size for binning and LightGBM (21) as the classification model and the S. aureus cases at CMUH from 2018 to 2019 as the training data set. The developed model was evaluated prospectively on the 2020 to 2021 CMUH data set and the four external data sets obtained from other hospitals. The MRSA/MSSA classification performance was evaluated using the AUC, sensitivity (true positive rate), and specificity (true negative rate) measures. Furthermore, we also developed a pseudo-gel plot module by using Python for data visualization of a large data set.

### Purification of the MRSA marker protein.

To identify a protein peak of *m/z* 6,593.2 and 6,550 Da, the proteins were extracted from a MRSA isolate and a MSSA isolate obtained from blood, respectively, as previously described (22). The extracted proteins were reconstituted in 0.1% FA and injected onto an ultraperformance liquid chromatography (UPLC) system (UltiMate 3000 RSLCnano system, Dionex, Amsterdam, the Netherlands) equipped with a liquid chromatography (LC) column (Waters Xbridge Protein BEH C4, 3.5 μm, 2.1 × 250 mm) for protein separation. The mobile phases were water (0.1% FA, phase A) and acetonitrile (ACN) (phase B). A 30-min gradient elution flowing at 250 μL/min was set as follows: 20% to 50% B for 22 min, 50% to 70% B for 2 min, 70% B for 3 min, and 70% to 20% B for 3 min. Thirty fractions were collected at 1 min per fraction. A 1-μL aliquot of each fraction was deposited onto a MALDI plate (Bruker Daltonics, Germany) and allowed to air dry. A 1-μL aliquot of the CHCA matrix solution (2 mg/mL, 50% ACN/0.1%TFA) was overlaid on the sample spot and then air dried. MALDI–TOF MS (Ultraflex III TOF/TOF; Bruker Daltonics, Germany) was operated in the linear positive-ion mode using a 25-kV accelerating voltage with a laser frequency of 50 Hz within the mass range 1,000 to 12,000 Da. The MALDI–TOF mass spectra were processed using flexAnalysis 3.4 software (Bruker Daltonics, Germany). Each fraction was analyzed using MALDI–TOF MS to confirm the presence of the marker peaks at *m/z* 6,550 and *m/z* 6,591. The purified peptides and proteins were identified via in-solution digestion and nanoLC–MS/MS analysis.

### In-solution digestion.

The MRSA fractions containing the peak of *m/z* 6,551 or *m/z* 6,591 and the parallel MSSA fractions were dried via vacuum centrifugation and then dissolved in 25 μL of a 50 mM ammonium bicarbonate (ABC) buffer (pH 8.5), followed by reduction using 10 mM dithiothreitol (DTT, 1 h) and alkylation in the dark using 55 mM iodoacetamide (IAA, 1 h). The alkylation reaction was quenched using 40 mM DTT for 1 h. The samples were separately digested overnight in sequencing-grade endoprotease Glu-C (Sigma-Aldrich, St. Louis, MO, USA) and trypsin (Promega, WI, USA) at 37°C and evaporated via vacuum centrifugation. The digested samples were resuspended in 10 μL of 0.1% FA before nanoLC–MS/MS.

### NanoLC–MS/MS and data analysis.

NanoLC–MS/MS was performed using a nanoflow UPLC system (UltiMate 3000 RSLCnano System, Dionex, Amsterdam, the Netherlands) coupled with a hybrid quadrupole TOF mass spectrometer (maXis impact, Bruker Daltonics, Germany) by employing a CaptiveSpray ion source. Digested peptide samples were injected into a trap column (Acclaim PepMap C18, 5 μm 100 Å, 100 μm × 250 mm, Thermo Fisher Scientific, USA) at a flow rate of 10 μL/min for 4 min. The trapped peptides were then eluted and separated in an analytical column (Acclaim PepMap C18, 2 μm 100 Å, 75 μm × 25 cm, Thermo Scientific, USA) at a flow rate of 300 nL/min. Peptides were separated along an ACN/water gradient (1% to 40%) within 60 min. For data-dependent acquisition, the five most intense precursors with charges +2, +3, and +4 from each TOF MS scan (*m/z* 400 to 2,000) were dynamically selected for MS/MS scanning (*m/z* 50 to 2,000). The MS/MS data were processed using DataAnalysis software version 4.4 (Bruker Daltonics, Germany). The S. aureus proteome (11,178 sequences, 6/26/2020) was downloaded from UniProt for reference. The MASCOT search parameters were as follows: fixed modification of carbamidomethylation (C), variable modification of oxidation (M), and deamidation (NQ), trypsin or Glu-C enzyme cleavage specificity, and mass-error tolerances of 50 ppm and 0.05 Da for the precursor and fragment ions, respectively. The false discovery rate (FDR) was set to 1%. Peptides were identified if their MASCOT individual ion scores were higher than 25.

### Data availability.

The de-identified materials supporting the findings of this study are available from the corresponding author upon request. The MALDI–TOF MS data generated in this study have been deposited in the Zenodo database available at https://zenodo.org/record/5502292#.YUXjRWaA6dY.
