# Supplementary material for: Rapid Identification of Methicillin-Resistant Staphylococcus aureus Using MALDI-TOF MS and Machine Learning from over 20,000 Clinical Isolates
Source: Microbiol Spectr. 2022 Mar 16;10(2):e00483-22. doi: 10.1128/spectrum.00483-22 (PMC9045122; doi:10.1128/spectrum.00483-22)
Supplement: SUPPLEMENTAL FILE 1 — Supplemental material. Download SPECTRUM00483-22_Supp_1_seq4.pdf, PDF file, 3.0 MB [file spectrum00483-22_supp_1_seq4.pdf]

Supplementary Table 1. F_1 scores of different combinations of bin sizes and ML algorithms. The combination of 10-Da bins and Light Gradient Boosting Machine has highest performance in the CMUH training dataset.

**F_1 Score**	**Bin Size (Da)**				
**ML Algorithms**	**1**	**5**	**10**	**15**	**20**
LightGBM	0.8131	0.8121	0.8134	0.8086	0.8026
Gradient Boosting	0.7985	0.7962	0.8017	0.7952	0.7899
Logistic Regression	0.8036	0.7971	0.7973	0.7866	0.7801
Extreme Gradient Boosting	0.7943	0.8045	0.8020	0.7916	0.7900
Extra Trees	0.7688	0.7648	0.7702	0.7634	0.7700
Random Forest	0.7629	0.7462	0.7583	0.7589	0.7596
Linear SVM	0.7728	0.7891	0.7790	0.7568	0.7504
Decision Tree	0.7294	0.7264	0.7219	0.7209	0.7214
K Neighbors	0.6802	0.7001	0.7164	0.7009	0.7259
Naive Bayes	0.6775	0.6793	0.6770	0.6782	0.6783

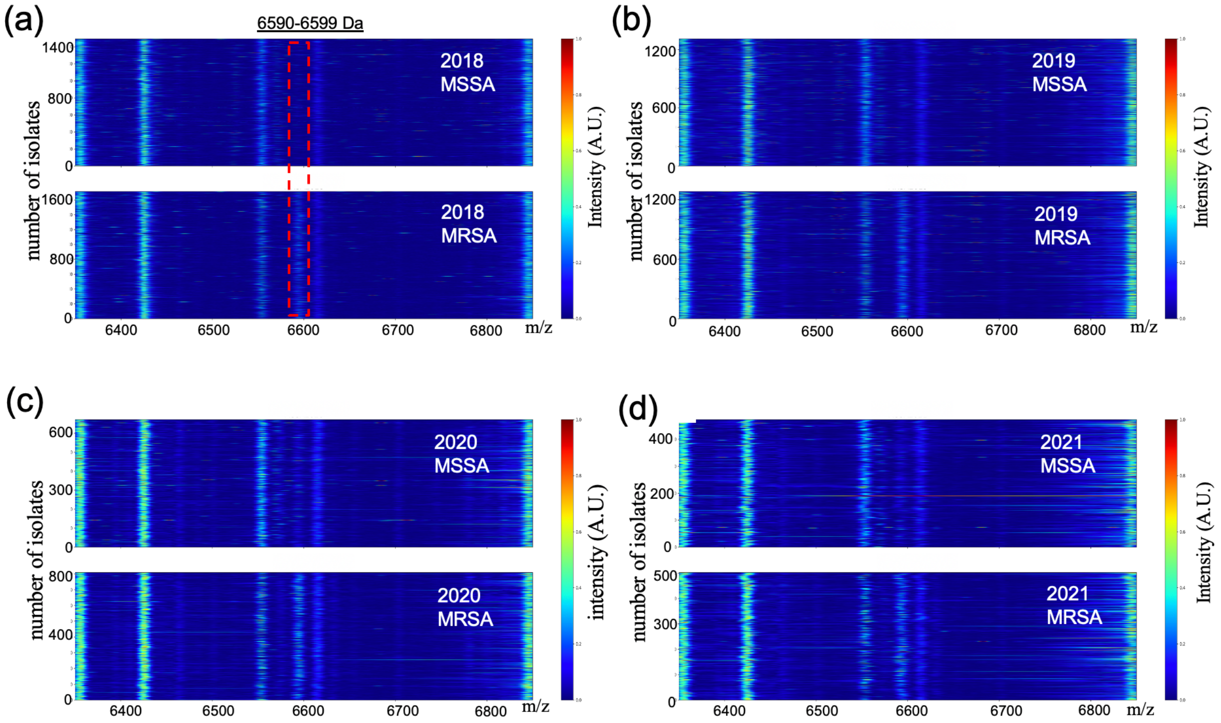

Supplementary Figure 1. Observations of the molecular feature at 6590–6599 Da in (a) year 2018 (b) year 2019 (c) year 2020 (d) year 2021

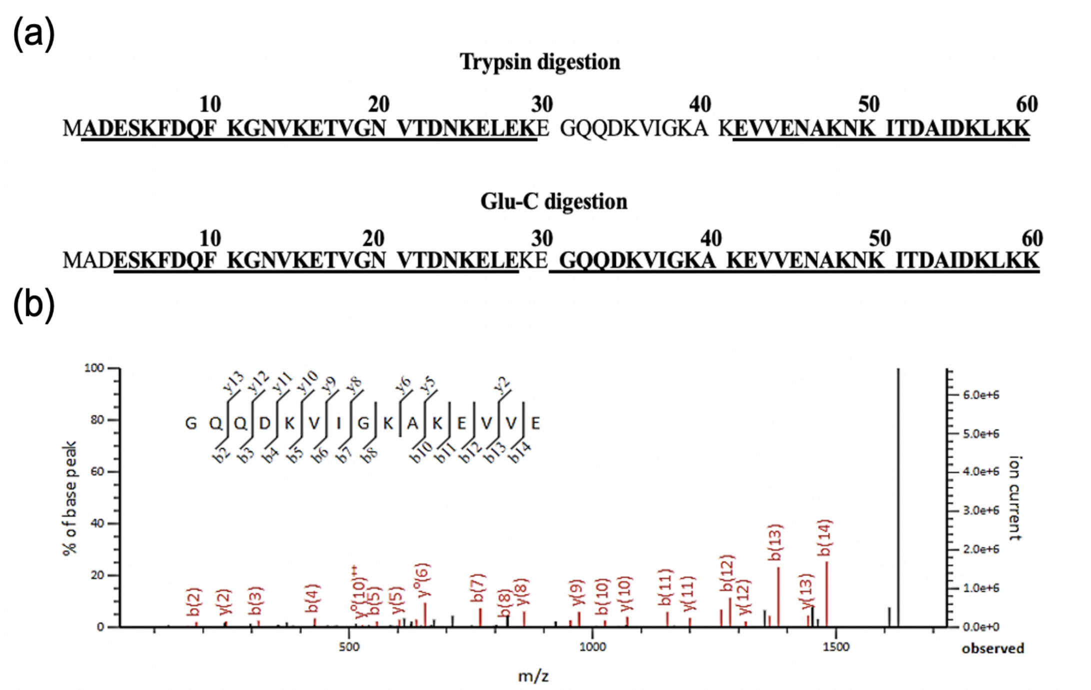

Supplementary Figure 2. The peak of m/z = 6593.2 in the MRSA strain was identified as UPF0337 protein SACOL1680. (a) The sequence of peptides separately identified in trypsin and Glu-C digestion was underlined and bolded. (b) The fragmented ion spectra of a peptide sequence of ^{31}GQQDKVIGKAKEVVE^{45}.

Supplementary Table 2. The identified peptide sequence of UPF0337 protein SACOL1680 and UPF0337 protein SA1452 by Trypsin and Glu-C. enzyme digestion and nanoLC-MS/MS analysis.

Sequence	Range	Scores	m/z meas.	Mr calc.	MH+ meas.	Rt [min]	Δ m/z [ppm]	Modifications
**UPF0337 protein SAUSA300 (MRSA_Fraction 7_Trypsin)**								
K.AKEVVENAK.N	40 - 48	34.6	494.261	986.5397	987.5147	13.18	-32.59	
K.ITDAIDKLK.K	51 - 59	38.0	508.789	1015.5914	1016.5707	15.36	-27.45	
K.NKITDAIDK.L	49 - 57	49.6	509.2692	1016.5502	1017.5312	14.19	-25.82	
K.ITDAIDKLKK.-	51 - 60	51.0	572.8394	1143.6863	1144.6715	13.65	-19.25	
M.ADESKFDQFK.G	2 - 11	89.7	607.7707	1213.5615	1214.5341	15.32	-28.54	
K.AKEVVENAKNK.I	40 - 50	56.2	615.3261	1228.6776	1229.645	11.01	-32.39	
K.NKITDAIDKLK.K	49 - 59	60.9	629.854	1257.7293	1258.7008	21.62	-28.38	
K.ETVGNVTDNKELEK.E	16 - 29	121.8	788.3759	1574.7788	1575.7444	13.91	-26.39	
M.ADESKFDQFKGNVK.E	2 - 15	121.2	806.8778	1611.7893	1612.7483	14.74	-29.89	
K.EVVENAKNKITDAIDK.L	42 - 57	143.6	893.955	1785.9472	1786.9027	18.83	-29	
**UPF0337 protein SAUSA300 (MRSA_Fraction 7_Glu-C)**								
E.TVGNVTDNKE.L	17 - 26	63.6	538.7546	1075.5146	1076.5019	13.25	-18.47	
E.NAKNKITDAIDKLKK.-	46 - 60	131.6	567.3288	1698.9992	1699.9719	15.07	-20.3	
E.NAKNKITDAIDKLKK.-	46 - 60	152.7	567.6577	1699.9832	1700.9584	18.62	-18.86	Deamidated: 1
E.NAKNKITDAIDKLKK.-	46 - 60	122.7	567.6578	1699.9832	1700.9588	17.07	-18.59	Deamidated: 4
D.KVIGKAKEVVE.N	35 - 45	35.2	600.3589	1198.7285	1199.7105	12.79	-21.1	

E.GQQDKVIGKAKE.V	31 - 42	88.5	650.8508	1299.7147	1300.6943	7.63	-21.26	
E.TVGNVTDNKELE.K	17 - 28	61.3	659.8144	1317.6412	1318.6215	15.34	-20.44	
E.SKFDQFKGNVKE.T	5 - 16	71.9	713.8551	1425.7252	1426.7029	13.37	-20.73	
E.KEGQQDKVIGKAKE.V	29 - 42	59.9	779.4154	1556.8522	1557.8235	8.86	-23.12	
E.GQQDKVIGKAKEVVE.N	31 - 45	99.1	814.4373	1626.8941	1627.8674	14.58	-20.86	
**UPF0337 protein SA1452 (MSSA_Fraction 5_Trypsin)**								
K.ITDAIDKLK.K	51 - 59	34.6	508.7906	1015.5914	1016.5739	15.48	-24.35	
K.NKITDAIDK.L	49 - 57	48.8	509.2681	1016.5502	1017.5288	14.13	-28.16	
K.ITDAIDKLKK.-	51 - 60	42.0	572.8364	1143.6863	1144.6655	13.59	-24.54	
M.ADESKFDQFK.G	2 - 11	89.8	607.7706	1213.5615	1214.5339	15.44	-28.67	
K.NKITDAIDKLK.K	49 - 59	44.2	629.8538	1257.7293	1258.7002	21.66	-28.82	
K.ETVGNVTDNKELEK.E	16 - 29	121.5	788.3747	1574.7788	1575.7421	13.93	-27.88	
K.ETVGNVTDNKELEK.E	16 - 29	31.0	788.8777	1575.7628	1576.7481	13.88	-13.91	Deamidated: 5
M.ADESKFDQFKGNVK.E	2 - 15	116.9	806.8795	1611.7893	1612.7516	14.28	-27.83	
K.EVVENAKNKITDAIDK.L	42 - 57	136.5	596.3058	1785.9472	1786.9027	18.99	-28.97	
**UPF0337 protein SA1452 (MSSA_Fraction 5_Glu-C)**								
E.TVGNVTDNKELE.K	17 - 28	52.6	659.8147	1317.6412	1318.6221	16.04	-19.97	
E.SKFDQFKGNVKE.T	5 - 16	72.7	713.8469	1425.7252	1426.6865	14.61	-32.22	
D.ESKFDQFKGNVKE.T	4 - 16	78.0	778.367	1554.7678	1555.7267	14.57	-31.1	
E.GQQDKATGKAKEVVE.N	31 - 45	31.7	794.3926	1586.8264	1587.778	6.74	-35.05	

E.NAKNKITDAIDKLKK.-	46 - 60	112.7	850.4794	1698.9992	1699.9515	14.96	-32.32	
E.NAKNKITDAIDKLKK.-	46 - 60	86.6	850.9701	1699.9832	1700.9329	17.57	-33.85	Deamidated: 4
E.NAKNKITDAIDKLKK.-	46 - 60	112.8	567.6513	1699.9832	1700.9392	18.49	-30.13	Deamidated: 1
